# Transcranial color-coded duplex sonography assessment of cerebrovascular reactivity to carbon dioxide: an interventional study

**DOI:** 10.1186/s12883-021-02310-9

**Published:** 2021-08-07

**Authors:** Stephanie Klinzing, Federica Stretti, Alberto Pagnamenta, Markus Bèchir, Giovanna Brandi

**Affiliations:** 1 Institute for Intensive Medicine, University Hospital of Zurich, Raemistrasse 100, CH-8091 Zurich, Switzerland; 2 Intensive Care Unit, Westmead Hospital, Westmead, NSW Australia; 3 Intensive Care Unit, Regional Hospital of Mendrisio, Mendrisio, Switzerland; 4 Unit of Clinical Epidemiology, Ente Ospedaliero Cantonale, Bellinzona, Switzerland; 5 Division of Pneumology, University of Geneva, Geneva, Switzerland

**Keywords:** Transcranial color-coded duplex sonography, Intensive care ultrasound, CO2 reactivity, Traumatic brain injury, Cerebral blood flow measurements

## Abstract

**Background:**

The investigation of CO_2_ reactivity (CO_2_-CVR) is used in the setting of, e.g., traumatic brain injury (TBI). Transcranial color-coded duplex sonography (TCCD) is a promising bedside tool for monitoring cerebral hemodynamics. This study used TCCD to investigate CO_2_-CVR in volunteers, in sedated and mechanically ventilated patients without TBI and in sedated and mechanically ventilated patients in the acute phase after TBI.

**Methods:**

This interventional investigation was performed between March 2013 and February 2016 at the surgical ICU of the University Hospital of Zurich. Ten volunteers (group 1), ten sedated and mechanically ventilated patients (group 2), and ten patients in the acute phase (12–36 h) after severe TBI (group 3) were included. CO_2_-CVR to moderate hyperventilation (∆ CO_2_ -5.5 mmHg) was assessed by TCCD.

**Results:**

CO_2_-CVR was 2.14 (1.20–2.70) %/mmHg in group 1, 2.03 (0.15–3.98) %/mmHg in group 2, and 3.32 (1.18–4.48)%/mmHg in group 3, without significant differences among groups.

**Conclusion:**

Our data did not yield evidence for altered CO_2_-CVR in the early phase after TBI examined by TCCD.

**Trial registration:**

Part of this trial was performed as preparation for the interventional trial in TBI patients (clinicaltrials.gov NCT03822026, 30.01.2019, retrospectively registered).

## Background

Cerebral autoregulation allows the maintenance of stable cerebral blood flow (CBF) despite changes in cerebral perfusion pressure (CPP) through variations of cerebral vascular resistance (CVR) [25]. Carbon dioxide (CO_2_) is a potent cerebral vasodilator, with a sigmoid relationship between paCO_2_ (arterial carbon dioxide) and CBF that can be assumed to be linear during acute changes in normophysiologic states [7] and which is mediated by CO_2_ -related changes in extracellular pH. This CO_2—_induced mechanism is commonly used in the clinical setting to reduce elevated intracranial pressure (ICP) by application of hyperventilation (HV), leading to hypocapnia. A decrease in paCO_2_ leads to a reduction in CBF, thus reducing cerebral blood volume, and, consequently, ICP. Changes in CVR and CBF in response to changes in CO_2_ are termed cerebrovascular reactivity to CO_2_ (CO_2_-CVR).

Several invasive and non-invasive techniques are currently available to assess CBF. These include, e.g., arterial and jugular venous tracer-concentration measurements (Kety-Schmidt method), Xenon clearance technique, positron emission tomography, near-infrared spectroscopy (NIRS), and transcranial Doppler (TCD). The choice of technique is dependent on the clinical scenario. The non-invasive bedside ultrasonography technique of TCD is an attractive tool for determining CBF and CO_2_-CVR. Reference values for CO_2_-CVR assessed by TCD in healthy volunteers are reported to range between 2.9 and 3.7%/mmHg [9, 11, 12, 16, 29]. For patients under general anesthesia, however, the potential effect of anesthetic agents has to be taken into account. Current data suggest maintained CO2-CVR during anesthesia and generally accepted values of 2.5–6% change in cm/s/mmHgCVR for CO_2_-CVR have been reported [5, 8, 15, 19, 27, 28]. In TBI, cerebral circulation may be compromised after injury. Data suggest that CO_2_-CVR may be preserved or impaired at various stages of TBI [12, 14, 21, 24]. Research concerning the association of impaired CO_2_-CVR and neurological outcome is ongoing, because conflicting results have been reported [3, 24].

Transcranial color-coded duplex sonography (TCCD) is an ultrasound technique, combining Doppler and Duplex effects, thus allowing the visualization of the examined vessels. As TCCD is more observer- independent than TCD [18], it could be an attractive tool for serial bedside measurements of flow velocities in the Intensive Care Unit (ICU) setting.

In the present interventional study, TCCD was used for assessing CO_2_-CVR. A systematic investigation of CO_2_-CVR by TCCD in healthy volunteers, patients on mechanical ventilation, and patients with TBI was conducted to investigate whether there is evidence for altered CO_2_-CVR in the acute phase of TBI in our study population.

## Methods

This study was conducted as an interventional trial in the surgical ICU of the University Hospital of Zurich between March 2013 and February 2016. The Cantonal Ethics Committee of Zurich approved and registered the study (KEK-ZH 2012–0542). Informed written consent was obtained from all participants or next of kin prior to study enrollment and/or from the patient after ICU discharge.

Patients in the TBI group were included in a study focusing on the effect of moderate hyperventilation on cerebral metabolism and thus selected according to previously published inclusion criteria [2]. Part of this trial was performed as preparation for the interventional trial in TBI patients (clinicaltrials.gov NCT03822026, retrospectively registered).

### Patient population

The study was conducted in spontaneously breathing volunteers (Group 1), sedated and mechanically ventilated patients with presumed preserved CO_2_-CVR (Group 2), and sedated and mechanically ventilated patients suffering from severe TBI (TBI Group 3).

Inclusion criteria for Group 3 were adults (≥ 18 years of age) with non-penetrating head injury, with an initial Glasgow Coma Scale (GCS) score < 9 prior to sedation and intubation, extended neuromonitoring with ICP, brain tissue oxygenation (P_br_O_2_), and/or microdialysis probes (TBI group), and also undergoing invasive mechanical ventilation with FIO_2_ < 60% and PEEP < 15 cmH_2_O. Exclusion criteria for all groups were decompressive craniectomy, pregnancy, pre-existing neurologic disease, previous TBI, acute cardiovascular disease, severe respiratory failure, acute or chronic liver disease, sepsis, and failure to obtain satisfactory bilateral TCCD signals. Patients with persisting hypovolemia or hemodynamic instability despite previous fluid resuscitation (defined as Global End-Diastolic Volume Index < 680 ml/m^2^, central venous oxygen saturation (ScvO_2_) < 60% and/or increase in mean arterial blood pressure (MAP) > 15% after passive leg raising test) were excluded.

The study was performed in the acute phase (12–36 h) after severe TBI (Group 3), while patients in Group 2 were investigated within 36 h after onset of mechanical ventilation.

All TBI patients were treated according to a cerebral perfusion orientated protocol aiming to achieve CPP > 70 mmHg, ICP ≤ 20 mmHg, P_br_O_2_ > 15 mmHg, P_a_CO_2_ between 4.8 and 5.2 kPa. For Group 2, a MAP of 65 mmHg was targeted.

### TCCD measurements

TCCD examination of the middle cerebral artery (MCA) was performed bilaterally via the transtemporal acoustic window by two experienced investigators (GB, SK), following standard techniques using a 5–1 MHz Probe (Philips CX 50, USA) [17]. Three repeated measurements of the peak systolic (PSV) and end-diastolic (EDV) velocity were performed for each side and an average value was calculated. The device also automatically calculated CBF-velocity (CBFV) and pulsatility index (PI).

### Study protocol

**Fig. 1:**
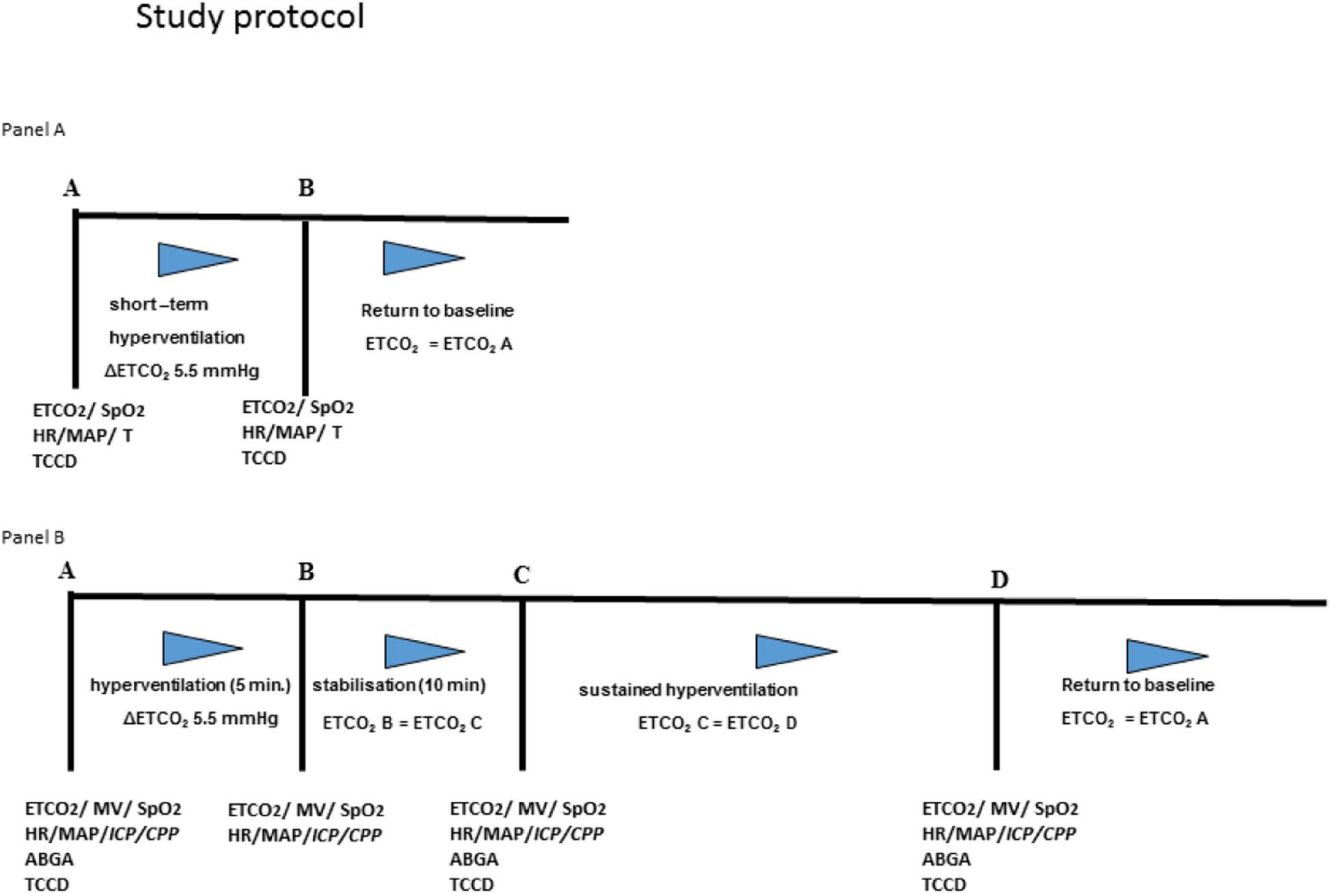
Study protocol. Panel **A**. Study protocol for group 1. During baseline conditions (**A**) and after short-term hyperventilation (**B**) the following parameters were recorded: end-tidal CO_2_ (ETCO_2_), peripheral capillary oxygen saturation (SpO_2_), heart rate (HR), mean arterial pressure (MAP), and auricular temperature (T). Measurements with transcranial color-coded duplex sonography (TCCD) were performed at time points **A** and **B**. Panel **B**. Study protocol for groups 2 and 3. During baseline conditions (**A**), after short-term hyperventilation (**B**), stabilization (**C**), and sustained hyperventilation (**D**) several parameters were recorded. ETCO_2_ remained stable during **B**, **C**, and **D**. TCCD measurements were performed during **A**, **C**, and **D**. An arterial blood gas analysis (ABGA) was obtained during **A**, **C**, and **D**. Only for patients in group 3 were values for intracranial pressure (ICP) and cerebral perfusion pressure (CPP) noted during **A**, **B**, **C**, and **D**. MV: minute volume

In Group 1, ten spontaneously breathing volunteers were examined (Fig. 1, Panel A) using end-tidal carbon dioxide (EtCO_2_) to monitor ventilation. Subsequently, each volunteer was asked to gradually increase respiratory rate and tidal volume to achieve a reduction in EtCO_2_ of approximatively 5.5 mmHg. Once the desired ∆ETCO_2_ was achieved, the volunteer maintained a stable minute ventilation and EtCO_2_ for the duration of the TCCD measurements. After the TCCD measurements, the volunteer returned to resting ventilation.

Ten sedated and mechanically ventilated ICU patients in Group 2 and ten patients with severe TBI in Group 3 were investigated (Fig. 1, Panel B).

Under baseline conditions, a TCCD examination was performed and all variables were recorded (Fig. 1, point A). The minute ventilation was then increased over a 10-min period to obtain moderate HV by a stepwise increase in tidal volume and respiratory rate until a reduction of EtCO_2_ of 0.7 kPa (Fig. 1, point B) was achieved.

After 10 min of stable EtCO_2_, a second TCCD measurement was undertaken (begin of HV, Fig. 1, point C). The EtCO_2_ value was kept stable for 40 min, and then followed by a third TCCD examination (Fig. 1, point D). Finally, normoventilation was re-established over 10 min and all variables were allowed to return to baseline (Fig. 1, point E). A final TCCD examination was conducted at this time point. At each time point, MAP, SpO_2_ and EtCO_2_ were recorded.

Arterial blood gas tests (ABG) were obtained at points A, C, D and E, to monitor the changes in pH and P_a_CO_2_.

For study purpose, measurements and values obtained at timepoint A and B was used for group 1, while timepoint A and D was used for group 2 and 3.

### Definition of cerebrovascular reactivity to carbon dioxide

CO_2_-CVR is expressed in terms of absolute and relative reactivity. Absolute CO_2_-CVR is defined as change in MFV (cm/s) per mmHg change in CO_2_. Relative CO_2_-CVR is defined as percentage change compared to baseline value.

$$\text{Absolute}\; \text{CO}_{2}-\text{CVR} = \Delta\text{MFV}/\Delta \text{CO}_{2}$$

$$\text{Relative}\; \text{CO}_{2}-\text{CVR} = ( \text{Absolute}\; \text{CO}_{2}-\text{CVR} / \text{baseline}\; \text{MFV}) \times 100$$

As the relative reactivity is less dependent on baseline values, it has been proposed as a more valuable indicator of CO_2_-CVR for analysis [10]. Relative reactivity was therefore chosen as the indicator for CO_2_-CVR.

∆MFV = difference in MFV between baseline and after HV.

∆CO_2_ = difference in CO_2_ between baseline and after HV. In Group 1, EtCO_2_ was used, while PaCO_2_ was used in Group 2 and TBI Group 3.

Hyperventilation constricts distal vessels, so a decrease in the absolute value of MFV is expected is the major intracranial vessels, as the ones investigated by TCCD.

### Statistical analysis

Descriptive statistics were presented as mean with standard deviation (SD) or as median with interquartile range (IQR) for quantitative data. Categorical data were presented as absolute numbers with percentages. Comparisons of continuous variables among the three groups were performed with one-way analysis of variance or with the Kruskal–Wallis-test, as appropriate. For statistically significant *p*-values, post-hoc tests were performed, taking the multiple comparisons into account. Qualitative data among the three groups were compared with the Chi-Square test. In cases of statistically significant results, post-hoc comparisons were made with the appropriate critical level adjustment. Comparisons of quantitative data before and during hyperventilation were conducted with the paired Student’s *t*-test or with the Wilcoxon matched pairs test, as appropriate. All tests were done two-sided, and *p*-values < 0.05 were considered statistically significant. Stata version 12.1 (StatCorp. LP, College Station, TX, USA) was used for all statistical analysis.

## Results

Baseline characteristics of Group 1, Group 2 and Group 3 are presented in Table 1. As stated in exclusion criteria, patients and volunteers included did not have comorbidities with known impact on cerebral autoregulation. Patients included in group 2 were admitted to the ICU after surgical care (Otolaryngoly (*n* = 3), plastic surgery (*n* = 2), thoracic surgery(*n* = 2), visceral surgery(*n* = 3)). Patients in Group 3 were under higher dosages of midazolam (*p* < 0.001), propofol (*p* = 0.004), fentanyl (*p* = 0.02), and norepinephrine (*p* = 0.008) compared to Group 2, while groups were comparable according to age, sex and BMI.

**Table 1:** Baseline characteristics

Parameter	Group 1 *n* = 10	Group 2 *n* = 10	Group 3 *n* = 10	p
Age (years)	34 ± 7.5	44 ± 16.3	35 ± 13.8	0.21
Sex (men)	8	4	7	0.16
BMI	25.4 ± 3.7	23.1 ± 2.5	24.0 ± 2.7	0.25
SAPS II	n.a	27 ± 11	47 ± 8	< 0.001
GCS	n.a	n.a	5.9 ± 2.8	n.a
ISS	n.a	n.a	30 ± 11	n.a
Depth of insonation MCA (mm)				
Right	47 ± 5	52 ± 4	54 ± 5	0.03^#^
Left	48 ± 4	51 ± 6	52 ± 5	0.10
Angle of insonation (°)				
Right	22 ± 10	36 ± 8	28 ± 12	0.01^*^
Left	25 ± 14	33 ± 13	25 ± 8	0.26
Midazolam (mg/kg/h) (n)	n.a	0.0 ± 0.0 (0)	0.3 ± 0.3 (7)	0.002
Propofol (mg/kg/h) (n)	n.a	4.3 ± 1.0 (10)	2.0 ± 1.8 (6)	0.003
Remifentanil (mg/kg/h) (n)	n.a	2.3 ± 2.3 (6)	1.5 ± 2.2 (4)	0.4
Fentanyl (mg/kg/h) (n)	n.a	0.1 ± 0.4 (3)	2.8 ± 3.0 (6)	0.02
Norepinephrine (µcg/kg/min)(n)	n.a	0.10 ± 0.06 (9)	0.28 ± 0.17(9)	0.004

Values are expressed as mean (standard deviation) or n as appropriate

*n.a.* not applicable, *BMI* body mass index, *SAPS II* simplified acute physiology score II, *GCS* Glasgow Coma scale; ISS- Injury Severity Score; MCA: middle cerebral artery

^*^ between Group 1 and 2. ^#^ between Group 1 and 3

All patients included to group 3 showed traumatic subarachnoidal hemorrhage on the initial CT scan. Seven patients showed bilateral contusional hemorrhage and three patients predominantly left sided contusional hemorrhage. Seven Patients were classified as Marshall 2, one patient as Marshall 3, one patient as Marshall 5 and two patients as Marshall 6.

While HR remained stable in all groups, MAP was significantly different between Group 1 and Group 2 (*p* = 0.001 and *p* = 0.008) and between Group 2 and Group 3 (*p* = 0.001 and *p* = 0.005) at baseline and during HV. HV lead to a significant increase in MV and corresponding decrease in EtCO_2_ and P_a_CO_2._ as well as a significant reduction of MFV in the right and left MCA in all groups (Table 2). Baseline MFV did not differ significantly between group 2 and 3, but was significantly higher at baseline in group 3 compared to group 1 (*p* = 0.024 (right), *p* = 0.032 (left)).

**Table 2:** Physiological data

	Group 1			Group 2			Group 3		
Parameter	Baseline	Short-term Hyperventilation	p	Baseline	Sustained Hyperventilation	p	Baseline	Sustained Hyperventilation	p
HR (b/min)	77 ± 14	78 ± 14	0.41	74 ± 18	75 ± 21	0.84	73 ± 17	72 ± 18	0.61
MAP (mmHg)	93 ± 8^1^	93 ± 8^2^	0.94	76 ± 11^3^	78 ± 12^4^	0.62	93 ± 9	94 ± 11	0.60
MV (l/min)	n.a	n.a	-	6.4 ± 1.7	9.2 ± 2.7	< 0.001	7.1 ± 1.4	8.9 ± 1.7	0.0037
ETCO_2_ (mmHg)	38.6 ± 3.7	30.8 ± 2.4	< 0.001	41.4 ± 6.9	34.4 ± 7.1	< 0.001	37.5 ± 5.5	31.8 ± 4.8	< 0.001
pH	n.a	n.a	-	7.36 ± 0.05	7.42 ± 0.05	< 0.001	7.37 ± 0.09	7.45 ± 0.02	< 0.001
paCO_2_ (mmHg)	38.5 ± 3.6*	30.6 ± 2.4*	< 0.001	39.3 ± 4.6	33.3 ± 4.4	< 0.001	37.4 ± 5.5	31.8 ± 4.7	< 0.001
MFV MCA (cm/s)									
Right	55 ± 10^5^	45 ± 7	0.0004	63 ± 25	51 ± 18	0.03	78 ± 22	65 ± 18	< 0.001
Left	55 ± 11^6^	47 ± 9	0.0003	65 ± 21	56 ± 14	0.04	78 ± 22	62 ± 12	0.005

Values are expressed as mean ± standard deviation

*HR* heart rate, *MAP* mean arterial pressure, *MV* minute ventilation, *ETCO*_*2*_ end-tidal carbon dioxide, *MFV* mean flow velocity, *MCA* middle cerebral artery, *n.a.* not applicable

^*^ Assumption of ETCO_2_ = paCO_2_

^1^*p* = 0.001 between group 1 and 2; ^2^*p* = 0.008 between group 1 and 2; ^3^*p* = 0.001 between group 2 and 3; ^4^*p* = 0.005 between group 2 and 3; ^5^*p* = 0.024 between group 1 and 3; ^6^*p* = 0.032 between group 1 and 3

Absolute and relative values for CO_2_-CVR for all groups are presented in Table 3. CO_2_-CVR was 2.14 (1.20–2.70) %/mmHg in group 1, 2.03 (0.15–3.98) %/mmHg in group 2, and 3.32 (1.18–4.48)%/mmHg in group 3.

**Table 3:** Cerebrovascular carbon dioxide reactivity

Characteristic	Group 1	Group 2	Group 3	p
Absolut CVR-CO_2_ ((cm/s) / mmHg)				
right	1.16 (0.76–1.78)	1.49 (0.15–3.26)	2.08 (1.22–3.49)	0.14
left	1.12 (0.52–1.58)	0.93 (-0.2–4.00)	2.12 (0.36–4.77)	0.38
overall	1.11 (0.71–1.58)	1.52 (0.02–3.21)	2.36 (0.80–4.34)	0.17
Relative CVR-CO_2_ (%/mmHg)				
right	2.25 (1.36–2.78)	2.07 (0.45–3.93)	2.64 (1.76–4.51)	0.4
left	2.15 (0.84–2.95)	1.49 (-0.53–4.5)	2.88 (0.51–5.48)	0.4
overall	2.14 (1.20–2.70)	2.03 (0.15–3.98)	3.32 (1.18–4.48)	0.28

Values are expressed as median (95%CI)

*CVR-CO*_*2*_ cerebrovascular carbon dioxide reactivity

Neither the CO_2_-CVR within-groups (comparison of the more- with the less-injured side) nor between-groups were significantly different.

## Discussion

### Main findings

The present study used TCCD to assess CO_2_-CVR in healthy volunteers, patients under sedation and mechanical ventilation without TBI and patients with severe TBI in the first 12–36 h after trauma. TCCD was conducted in the acute phase after TBI as part of another study. [2]

A relative CO_2_-CVR of 2.14%/mmHg (95% CI 1.20–2.70) was found in volunteers, 2.03%/mmHg ( 95% CI 0.15–3.98) in sedated and mechanically ventilated patients and 3.32%/mmHg (95% CI 1.18–4.48) in patients in the acute phase after TBI. CO_2_-CVR values between groups was not significantly different.

### How our data compare to the literature

In our TCCD study, relative CO_2_-CVR values in healthy volunteers 2.14%/mmHg (95% CI 1.20–2.70) were lower than those obtained by Klingelhofer et al.[12], which showed a mean CO_2_-CVR of 3.7 ± 0.5%/mmHg. Flow velocities obtained via TCCD might be higher than TCD values due to correction of the angle of incidence in TCCD measurements [1]. This may influence relative CO_2_-CVR when TCCD is used. For patients under general anesthesia undergoing major surgery, CO_2_-CVR assessed with TCD was reported to be preserved and mainly comparable with that of healthy volunteers [5, 8, 19, 27, 28]. This suggests a negligible influence of routinely used anesthetic agents on CO_2_-CVR. In our study, patients received intravenous analgosedation with Propofol and Remifentanil or Fentanil, in accordance to the referred studies, we did not find evidence of impact of those agents on CO_2_-CVR. Current values of CO2-CVR around 2.5–6% change in cm/s/mmHg are generally accepted [15]. In accordance with published data, we found a preserved CO_2_-CVR in our group of sedated and mechanically ventilated patients without TBI [5, 8, 19, 27, 28].

In our TBI patients, CO_2_-CVR was 3.32%/mmHg (95% CI 1.18–4.48). However, the increase in CO_2_-CVR did not reach statistical significance. Comparing our data with that in existing literature, some aspects deserve consideration. Klingelhofer et al. [12] reported a decreased but preserved CO_2_-CVR of 2.0 ± 1.1%/mmHg in 40 patients with acute traumatic and spontaneous cerebral hemorrhage, of whom 24 were in barbiturate coma. As barbiturates have been shown to influence CO_2_-CVR by metabolic suppression [23], this needs to be taken into account. CO_2_-CVR was reported to be preserved in other studies with TBI patients, although especially in the acute phase after TBI, impaired CO_2_-CVR was observed [14, 21, 24, 26].

In comparison with the cumbersome direct measurement of CBF, the non-invasive, bedside tool of sonography has the advantage of serial measurements of MFV and CO_2_-CVR in critically ill patients, although invasive and non-invasive methods complement each other, depending on the clinical scenario.

In our opinion, TCCD offers advantages compared to TCD in the daily setting of an ICU for non-continuous serial measurements, as it has been proven to be less operator dependent [18]. Furthermore, good reliability of interobserver results of TCCD measurements in TBI patients for trained operators has been reported, thus underscoring the value of TCCD to obtain reliable measurements [4]. This is an important aspect in the ICU setting, where serial measurements are performed by variably skilled operators. We were previously able to demonstrate a steep learning curve for residents introduced to TCCD in healthy volunteers [13]. Depending on the clinical scenario, TCCD seems to be interchangeable with TCD for serial monitoring of CO_2_-CVR, while TCD offers the advantage of continuous monitoring over time with a fixed probe.

TBI patients have been shown to have impaired cerebrovascular reactivity during long periods of their ICU stay, with a limited impact of current ICU treatment and an association of impaired cerebrovascular reactivity and outcome [6, 30]. Our study results do not suggest impaired CO_2_ – CVR. Of notice, CO_2_ – CVR is only one of several mechanism of cerebral autoregulation, thus preserved CO_2_-CVR does not imply intact cerebral autoregulation. While on the one hand it is known that prolonged HV can negatively affect outcome[20], on the other hand it has been postulated that hyperventilation, when CO2-CVR is intact, temporarily improves cerebral autoregulation[22]. Thus, our finding of preserved CO2-CVR in the early phase after TBI encourages that cautious hyperventilation under monitoring may be considered a therapeutic option [2]. Furthermore, TCCD may serve as a monitoring tool for serial assessment of CO2-CVR, which may change during the course of TBI, to detect signs of deterioration or recovery of CO2-CVR.

## Limitations

One limitation of this study is the small sample size; our results should be confirmed in larger studies of TBI patients. As well, the number of volunteers and patients examined in our number is too small to establish reference values. In a larger study, TCCD measurements for the assessment of CO2-CVR should be performed taking the localization of the insult into account. Furthermore, TCCD measurements for the assessment of CO_2_-CVR should be performed in both the early and later time course after trauma, taking the localization of the insult into account. Finally, a comparison of CO_2_-CVR obtained by TCCD and TCD would be desirable.

## Conclusion

Our data did not yield evidence for altered CO_2_-CVR in the early phase after TBI and TCCD a reliable tool for determination of CO_2_-CVR.

## Data Availability

The datasets used and analyses during the current study are available from the corresponding author on reasonable request.

## Abbreviations

ABGA: Arterial blood gas analysis
BMI: Body mass index
CO_2_-CVR: CO_2_ reactivity
CPP: Cerebral perfusion pressure
ETCO_2_: End-tidal carbon dioxide
HR: Heart rate
HV: Hyperventilation
ICU: Intensive care unit
MAP: Mean arterial pressure
MCA: Middle cerebral artery
MFV: Mean flow velocity
MV: Minute ventilation
SAPS II: Simplified acute physiology score II
TBI: Traumatic brain injury
TCD: Transcranial doppler sonography
TCCD: Transcranial color-coded duplex sonography
